# Mediating Role of Self‐Efficacy in Transition Shock and Presenteeism Among Nursing Students in China: A Cross‐Sectional Study

**DOI:** 10.1155/jonm/5586956

**Published:** 2026-09-18

**Authors:** Chanyi Jia, Yujia Wang, Siyu Chen, Xiaoxia Liu, Lina Ge, Shuang Zang

**Affiliations:** ^1^ Department of Obstetrics and Gynecology, Shengjing Hospital of China Medical University, Shenyang, China, cmu.edu.cn; ^2^ Department of Community Nursing, School of Nursing, China Medical University, Shenyang, China, cmu.edu.tw

**Keywords:** mediating effect, nursing student, presenteeism, self-efficacy, transition shock

## Abstract

**Background:**

Nursing students, as the reserve force of the nursing workforce, often experience transition shock during clinical internships, which negatively impacts their physical and mental health and may lead to presenteeism. Self‐efficacy, a modifiable psychological resource, offers a key intervention target for improving this situation.

**Objective:**

To examine self‐efficacy’s mediating role between transition shock and presenteeism in nursing students and to guide management.

**Methods:**

This study adopted a descriptive cross‐sectional design. A convenience sampling survey was conducted on 421 nursing students in Shenyang City, China. The questionnaire included demographic characteristics, the General Self‐Efficacy Scale (GSES), the Transition Shock Scale for Undergraduate Nursing Students (UNSTS), and Stanford Presenteeism Scale (SPS‐6). Descriptive statistics, correlation analysis, and multiple linear regression were performed using IBM SPSS 27.0. The structural equation model was used to test the mediating effect hypothesis of self‐efficacy between transition shock and presenteeism, and AMOS 28.0 was used to estimate the path coefficients and evaluate the model fit.

**Results:**

The research found that the presenteeism, transition shock, and self‐efficacy level of nursing students in Shenyang were all at a moderately high level. Transition shock showed statistically significant positive correlations with presenteeism (*r* = 0.646) and negative correlations with self‐efficacy (*r* = −0.576), while self‐efficacy was negatively correlated with presenteeism (*r* = −0.636). The SEM showed satisfactory model fit. The results indicated that self‐efficacy played a partial mediating role between transition shock and presenteeism (effect = 0.212, [0.149.0.294], *p* < 0.001), accounting for 29.04% of the total effect (total effect = 0.73, [0.615.0.823], *p* < 0.001).

**Conclusion:**

This study has confirmed that self‐efficacy plays a mediating role between the transition shock and presenteeism among nursing students in Shenyang City, China.

**Implication for Nursing Management:**

This study offers nursing managers a new way to address presenteeism among nursing students and a reference for improving clinical internship quality and talent cultivation models.

## 1. Background

The growing demand for highly qualified nurses is driven by global challenges such as population aging and the rising burden of chronic diseases [1, 2]. Clinical internships serve as a pivotal bridge for nursing students who were defined as undergraduate students enrolled in a bachelor’s degree program in nursing who were currently completing their clinical internship rotations in hospitals, facilitating the accumulation of clinical experience during their transition to registered nurses [3, 4]. However, when they transition from theoretical learning to clinical internships, they often encounter various difficulties, pressures, and shocks [5]. The concept of transition shock is used to describe the confusion, doubt, and unclear positioning experienced in the process of transforming from a student to a nurse in terms of physiology, psychology, knowledge and skills, and social and cultural development [6]. Nursing students often experience transition shock during the clinical transition period, which may harm their physical and mental health [7, 8]. When their physical and mental health is compromised, students should take leave to recuperate, but for various reasons, they still attend classes and work, thus exhibiting “presenteeism”—a state of working while ill but with low efficiency [9]. Thus, transition shock is likely a direct antecedent of presenteeism.

Presenteeism is a new concept describing the work behavior or attendance status of nurses. Presenteeism not only statistically significantly reduces the work initiative and engagement of nurses but also further leads to an increase in error rates, delayed responses, and insufficient communication during the nursing process, thereby directly damaging the quality and safety of nursing services [10]. Self‐efficacy refers to people’s confidence in their ability to utilize the skills they possess to complete a certain task in a specific task domain [11]. In the clinical context, we propose that students with low self‐efficacy are more prone to presenteeism, as they lack the confidence to actively engage in unfamiliar procedures or seek help from preceptors, instead resorting to passive “mental withdrawal” as an avoidance strategy [12, 13]. Research has found that self‐efficacy is a key psychological resource for improving presenteeism and an important protective factor [14].

Presenteeism has been well studied in practicing nurses, with evidence showing a high incidence rate and economic costs exceeding those of general sick leave [15, 16]. For those who have not yet officially entered the workplace and are in a critical formative stage, the phenomenon of “being physically present but mentally absent” during clinical internships not only affects their current learning outcomes but may also shape an unhealthy career model in the future, and its long‐term harm is worth considering [17]. Nursing students are at a crucial stage of transformation into nurses during their internships, and it is necessary to pay attention to this group [18]. Moreover, existing research mostly focuses on the direct paths of transition shock and presenteeism, neglecting the possible mediating role of self‐efficacy between the two [19]. Therefore, this study aims to deeply understand the current situation of presenteeism among nursing students and analyze its influencing factors and the mediating effect of self‐efficacy in the relationship between transition shock and presenteeism among nursing students during their internships.

This study is based on the Job Demands–Resources (JD–R) theory [20]. The transition shock experienced by nursing students during their internships is a dense and multidimensional “job demand” set, including role ambiguity, skill‐reality gap, emotional overload, and relationship reconstruction pressure, and others [21]. According to the JD–R framework, when these high demands persist and individuals are unable to effectively cope, it will severely deplete the limited personal psychological resources of nursing students. Among these, self‐efficacy—that is, an individual’s belief in their ability to successfully complete clinical nursing tasks—is one of the most crucial personal resources [22]. Damaged self‐efficacy, as a depleted personal resource, leads nursing students to more easily fall into the predicament of “working while sick” with low efficiency, even when they are in poor condition. In other words, the transition shock not only directly causes presenteeism but also indirectly aggravates it through the medium of undermining the self‐efficacy of nursing students. Therefore, this study positions self‐efficacy as the core mediating mechanism between the transition shock and presenteeism.

Based on the above findings, this study proposes four hypotheses.

**Hypothesis 1.**

Transition shock is positively correlated with presenteeism.

**Hypothesis 2.**

Transition shock is negatively correlated with self‐efficacy.

**Hypothesis 3.**

Self‐efficacy is negatively correlated with presenteeism.

**Hypothesis 4.**

In nursing students, self‐efficacy plays a mediating role between transition shock and presenteeism.

**FIGURE 1:**
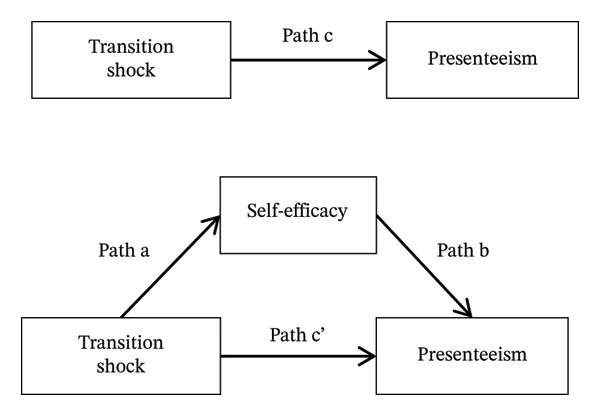
Hypothesis model figure.

The hypothesized model of this study is shown in Figure 1.

By exploring the mediating effect of self‐efficacy in the relationship between transition shock and presenteeism among nursing students and understanding the underlying mechanisms, this study aims to provide new ideas for optimizing the internship management process, improving presenteeism behavior, ensuring the quality of nursing talent cultivation, and facilitating the critical transition of nursing students from students to nurses.

## 2. Materials and Methods

### 2.1. Study Design

This study is a cross‐sectional study.

### 2.2. Participants

Using the convenience sampling method, a survey was conducted on nursing students who were in the internship period and met the inclusion and exclusion criteria from China Medical University, Liaoning University of Traditional Chinese Medicine, and Shenyang Medical College in Shenyang, China, from October 2025 to December 2025. The ranking criteria are as follows:

Inclusion criteria

1. Undergraduate nursing students in their final year of clinical internship;
2. Informed and voluntarily participating in this study;
3. Internship duration > 3 months.

Exclusion criteria

1. Those with mental or physical illnesses;
2. Those who have been absent from work due to illness or personal leave for more than 7 days cumulatively.

The primary outcome measure of this study is quantitative data, namely the level of presenteeism among nursing students. According to the sample size estimation method advocated by Kendall [23], the sample size should be at least 5 to 10 times that of the independent variable of the study. The general information questionnaire contains 12 variables, the General Self‐Efficacy Scale (GSES) has 1 dimension, and the transition impact evaluation scale for nursing undergraduate interns has 6 dimensions. Considering 20% invalid questionnaires, the sample size of this study should be at least 114 to 228. This study referred to the methodological guidelines proposed by Fritz and MacKinnon [24], and in the sample size design, fully considered the requirements of the mediation effect test, ensuring that the sample size could provide a statistical power greater than 0.8 to detect moderate and small indirect effects. We did not blindly assume that there would be an easily detectable large effect but adopted a more rigorous and demanding standard to plan our research, ensuring that even if the effect was not large, we still had sufficient confidence to discover it. This approach not only helps to enhance the generalizability of the research results but also effectively reduces the impact of sampling bias, which is a reasonable and relatively conservative expectation for the context of this study. The sample size of this article is 421, all meeting the above standards.

### 2.3. Data Collection

Before conducting the investigation, the consent of the school and the relevant internship hospitals was obtained first. Recruitment notices were distributed via WeChat through class advisors or monitors to nursing students. Eligible participants were provided with detailed information regarding the study purpose, procedures, confidentiality protections, and their right to withdraw at any time without academic or professional consequences. After confirming that all participants fully understood the study conditions and provided informed consent, an anonymous link to the Questionnaire Star was sent via WeChat. The questionnaire survey was distributed via Questionnaire Star, an online survey platform widely used in China. All responses were collected directly through the platform backend and subsequently exported by trained research staff. To enhance data quality, an attention‐check item was embedded in the questionnaire to identify and exclude invalid responses. We received a total of 437 questionnaires; after eliminating the questionnaires with incorrect answers to the traps, the number of valid questionnaires was 421, and the completion rate was 96.34%.

### 2.4. Measures

#### 2.4.1. Demographic Characteristics

By reviewing the literature, consulting experts, and combining with the research content, the general demographic data questionnaire for the nursing students in this study was determined. It includes 12 variables: age, gender, nationality, place of family residence, healthcare worker in family, experience as a student leader, self‐rated physical health status, self‐rated mental health status, method of nursing major selection, attitude toward the nursing profession, career intention, and night shift frequency per month.

#### 2.4.2. GSES

This scale was developed by Schwarzer et al. [25] and was translated and adapted into Chinese by Kangcai Wang et al. in 2001 [26]. It is used to measure an individual’s competence and confidence in coping with various challenges in different environments. The scale has one dimension and 10 items. Each item is scored from *completely incorrect* to *completely correct* on a scale of 1–4, with a total score ranging from 10 to 40. The higher scores indicate the higher levels of self‐efficacy. This scale is widely used among domestic nursing students. In this study, the Cronbach’s *α* coefficient of this scale is 0.890.

#### 2.4.3. Transition Shock Scale for Undergraduate Nursing Students (UNSTS)

This scale was developed by Kim et al. [27] in South Korea and was translated and adapted into Chinese by Yuxuan Huang et al. [28]. It is used to evaluate the degree of transition impact for undergraduate nursing students. The scale consists of 6 dimensions and 18 items, including 5 items on confusion of nursing professional values, 3 items on conflict between theory and practice, 3 items on tense interpersonal relationships, 3 items on excessive workload during internship, 2 items on incompatibility between clinical internship and personal life, and 2 items on lack of social support. The scale uses Likert 4‐point scoring, with scores ranging from *very disagree* to *very agree* on a scale of 1–4, and the total score ranges from 18 to 72. The higher the score, the greater the transition impact faced by the undergraduate nursing students. This scale has good reliability and validity among the nurse population. In this study, the Cronbach’s α coefficient is 0.874.

#### 2.4.4. Stanford Presenteeism Scale (SPS‐6)

This scale was developed by Koopman et al. [29] and was translated and revised by Chinese scholars Fang Zhao et al. [30] to form the Chinese version. This study employed the Chinese version of the scale. It is mainly used to measure an individual’s condition of being affected in work efficiency due to health problems (including physical and mental health) in the past period. The scale includes 2 dimensions: work limitation and work energy, with 6 items. These two dimensions correspond to the original English version’s “work process impact”’ and “work output impact” constructs, respectively, but were relabeled based on the empirical factor structure observed in the Chinese validation sample and adapted to Chinese linguistic conventions. Specifically, work limitation refers to the degree to which health problems restrict the employee’s ability to initiate, complete, or derive satisfaction from work tasks. Work energy refers to the perceived level of concentration and vitality available for work activities despite health issues. Each item uses Likert 5‐point scoring, ranging from *completely disagree* to *completely agree* on a scale of 1–5. The total score is the sum of the scores of the 6 items. Items 5 and 6 are reverse‐scored. The score range is 6–30. It should be noted that, during the cross‐cultural adaptation, Item 2 in the English version was rephrased from the original positively worded statement (“Despite having my health problem, I was able to finish hard tasks in my work”) to a negatively worded one to better conform to Chinese linguistic conventions and to maintain satisfactory statistical reliability. The Chinese version reads: “In the past month, due to health reasons, I was unable to finish difficult tasks in my work.” Overall, the scoring direction in the Chinese version is reversed relative to the English original. After our verification, a higher score on the Chinese version indicates greater productivity loss. In this study, the Cronbach’s *α* coefficient is 0.849.

### 2.5. Statistical Analysis

The data were statistically analyzed using SPSS 27.0 (SPSS Inc., Chicago, IL, USA) software. Firstly, descriptive statistical analysis was conducted on the demographic characteristics, with the results presented in terms of frequency and percentage. Descriptive statistical analysis was also performed on the main research variables (i.e., the three scales), and normality tests were carried out on the data. According to Byrne and Campbell, when the skewness and kurtosis values are close to zero (between −1.5 and + 1.5), the data can be considered to follow a normal distribution [31]. The data were all normally distributed or approximately normally distributed, and the results were presented in terms of mean and standard deviation. Secondly, Pearson correlation analysis was used to test the correlations among the main research variables. Next, the score of presenteeism of nursing students was taken as the dependent variable, and the independent variables included demographic characteristics, transition shock, and self‐efficacy. A multiple regression equation was constructed, and multiple regression analysis was used to explore the influencing factors of presenteeism among nursing students. Finally, AMOS 28.0 software was used to construct a structural equation model (SEM). The direct and mediating effects were estimated using the bias‐corrected Bootstrap resampling method, with 5000 iterations and a 95% confidence interval. This method was used to test the mediating effect of the hypothesis where presenteeism was the dependent variable, transition shock was the independent variable, and self‐efficacy was the mediating variable.

### 2.6. Ethics Approval

This study was conducted in accordance with the Declaration of Helsinki and approved by the Ethics Committee of Shengjing Hospital of China Medical University in Shenyang (2025PS1825K). All data were collected anonymously and kept confidential.

## 3. Results

### 3.1. Descriptive Statistics of Demographic Information

A total of 437 questionnaires were distributed in this study. After eliminating the invalid ones, 421 valid questionnaires were obtained, with an effective recovery rate of 96.34%. Other general information is shown in Table 1.

**TABLE 1:** Descriptive statistics of demographic information.

Variable	Category	*n*	%
Gender	Male	42	10.0
	Female	379	90.0

Age (years)	≤ 21	267	63.4
	22–23	134	31.8
	≥ 24	20	4.8

Nationality	Han nationality	319	75.8
	Man nationality	53	12.6
	Hui nationality	8	1.9
	Other	41	9.7

Place of family residence	Urban	278	66.0
	Other	143	34.0

Healthcare worker in family	Yes	106	25.2
	No	315	74.8

Experience as a student leader	Yes	254	60.3
	No	167	39.7

Self‐rated physical health status	Good	187	44.4
	Fair	166	39.4
	Average	59	14.0
	Poor	9	2.1

Self‐rated mental health status	Good	200	47.5
	Fair	164	39.0
	Average	48	11.4
	Poor	9	2.1

Method of nursing major selection	Personal interest	186	44.2
	Parental/others’ advice	179	42.5
	Program assignment	56	13.3

Attitude toward the nursing profession	Good	132	31.4
	Fair	192	45.6
	Average	87	20.7
	Poor	10	2.4

Career intention	Clinical nurse	255	60.6
	Further education	101	24.0
	Other	65	15.4

Night shift frequency per month	0	66	15.7
	1–5	156	37.1
	5–10	171	40.6
	≥ 10	28	6.7

### 3.2. Common Method Bias

Considering that the data of this study were all collected through questionnaires, in order to test for common method bias, the Harman single‐factor analysis method was adopted. After conducting an exploratory factor analysis without rotation on the scale, it was found that there were a total of 7 factors with eigenvalues greater than 1. Among them, the variance explained by the first factor was 32.326%, which was below the critical value of 40%. Therefore, there was no statistically significant common method bias in this study.

### 3.3. Descriptive Statistics of Presenteeism, Transition Shock, and Self‐Efficacy

Table 2 presents the descriptive statistics of presenteeism, transitional shock, and self‐efficacy of nursing students. The score for presenteeism is 18.38 ± 5.88. The level of presenteeism among nursing students is at an above‐average level. The score for the transitional shock is 49.21 ± 9.23. The level of transitional shock experienced by the nursing students is at an above‐average level. The score for self‐efficacy is 26.86 ± 5.98.

**TABLE 2:** Descriptive statistics of presenteeism, transition shock, and self‐efficacy.

Variable	Range	M	SD	Min	Max
Conflict between theory and practice	3–12	8.06	1.85	3	12
Overwhelming workload	3–12	9.06	1.82	4	12
Loss of social support	2–8	5.34	1.44	2	8
Diminishing relationship with a co‐worker	3–12	8.33	2.09	3	12
Confusion in professional nursing values	5–20	13.43	3.24	5	20
Incongruity in work and personal life	2–8	4.99	1.50	2	8
Transition shock	**18–72**	**49.21**	**9.23**	**22**	**72**
Self‐efficacy	**10–40**	**26.86**	**5.98**	**10**	**40**
Work limitations	4–20	12.25	4.06	4	20
Work energy	2–10	6.13	2.08	2	10
Presenteeism	**6–30**	**18.38**	**5.88**	**6**	**30**

*Note:* Bold values indicate the total scores of the main variables.

In the present study, because we excluded participants who had taken more than 7 days of sick leave, all remaining participants were physically present at work or clinical practice. Therefore, the crude prevalence of presenteeism—defined as an SPS‐6 total score greater than 6 (the minimum possible score)—was 100% in our analytical sample. To further illustrate the distribution of productivity loss, we also calculated the proportion of participants whose SPS‐6 total score exceeded the sample mean (*M* = 18.38, SD = 5.88), which was 48.22%. However, we acknowledge that there is currently no universally established clinical cutoff point for the Chinese version of the SPS‐6 to define a “case” of presenteeism; thus, we primarily treated the SPS‐6 as a continuous measure of functional impairment in our main analyses.

### 3.4. Correlations Among Presenteeism, Transition Shock, and Self‐Efficacy

The Pearson correlation analysis revealed statistically significant correlations among the key variables. As hypothesized, the transformation shock was statistically significantly positively correlated with presenteeism (*r* = 0.646). Conversely, there was a statistically significant negative correlation between the transformation shock and self‐efficacy (*r* = −0.576). There was also a statistically significant negative correlation between self‐efficacy and presenteeism (*r* = −0.636), as shown in Table 3.

**TABLE 3:** Correlations among presenteeism, transition shock, and self‐efficacy.

	Correlation results	Transition shock	Self‐efficacy	Presenteeism
Transition shock	Pearson	1	−0.576^∗∗^	0.646^∗∗^
	Sig.		< 0.001	< 0.001
	N	421	421	421

Self‐efficacy	Pearson	−0.576^∗∗^	1	−0.636^∗∗^
	Sig.	< 0.001		< 0.001
	N	421	421	421

Presenteeism	Pearson	0.646^∗∗^	−0.636^∗∗^	1
	Sig.	< 0.001	< 0.001	
	N	421	421	421

^∗∗^ *p* < 0.001.

### 3.5. Regression Analysis of Presenteeism

The regression model statistically significantly predicted nursing students’ presenteeism (*R* ^2^ = 0.626, adjusted *R* ^2^ = 0.601, *F* = 24.415, *p* < 0.001), with no multicollinearity concerns (all VIF < 3). Several demographic factors showed significant effects. Female gender negatively predicted presenteeism, while having healthcare workers in the family and having experience as a student leader were positive predictors. Regarding professional factors, compared to those with a good attitude toward the nursing profession, participants with an average attitude reported lower presenteeism. For career intention, compared to those aiming to become clinical nurses, participants planning further education or other careers reported higher presenteeism. Transition shock emerged as the strongest positive predictor, whereas self‐efficacy was the strongest negative predictor. Detailed results are presented in Table 4.

**TABLE 4:** Regression analysis of presenteeism.

Independent variable	B	SE	*β*	*t*	*p*	VIF	*R* ^2^	F
Constant	15.555	2.102		7.401	< 0.001		0.626	24.415
Gender	−2.343	0.643	−0.120	−3.641	< 0.001	1.134		
Healthcare worker in family	1.570	0.459	0.116	3.417	0.001	1.213		
Experience as a student leader	2.223	0.416	0.185	5.337	< 0.001	1.266		
Attitude toward the nursing profession—average	−1.843	0.626	−0.127	−2.943	0.003	1.962		
Career intention—further education	0.994	0.463	0.072	2.147	0.032	1.193		
Career intention‐other	2.269	0.579	0.140	3.919	< 0.001	1.335		
Transition shock	0.228	0.026	0.358	8.896	< 0.001	1.705		
Self‐efficacy	−0.336	0.039	−0.342	−8.552	< 0.001	1.682		

### 3.6. Mediating Effect of Self‐Efficacy on the Associations of Transition Shock and Presenteeism

**FIGURE 2:**
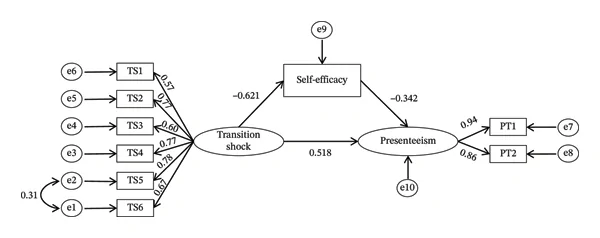
Structural equation model of the mediating effect of self‐efficacy between transition shock and presenteeism. TS1: conflict between theory and practice, TS2: overwhelming workload, TS3: loss of social support, TS4: diminishing relationship with a co‐worker, TS5: confusion in professional nursing values, TS6: incongruity in work and personal life, PT1: work limitations, PT2: work energy.

The SEM demonstrated a good fit with the data. The obtained fit statistics are as follows: *χ* ^2^/df = 2.902; NFI = 0.965; TLI = 0.965; IFI = 0.977; AGFI = 0.933; CFI = 0.977; GFI = 0.964; RMSEA = 0.067; and SRMR = 0.03, details can be found in Supporting Table S1. The path diagram and coefficients are presented in Figure 2. The standardized path coefficients of all direct and indirect effects are listed in Table 5. The Bootstrap analysis indicates that the transformation shock has a statistically significant indirect effect on presenteeism through self‐efficacy, accounting for 29.04% of the total effect. The direct effect of the transformation shock on presenteeism remains statistically significant. These findings imply that among Chinese intern nursing students, self‐efficacy plays a partial mediating role between the impact of transition shock and presenteeism.

**TABLE 5:** Mediating effect of self‐efficacy on the associations of transition shock and presenteeism.

Effects	Model paths	Estimate	SE	*p*	95% CI	Effect ratio
Direct effects	TS ⟶ SE	−0.621	0.041	< 0.001	[−0.695, −0.536]	
	SE ⟶ PT	−0.342	0.058	< 0.001	[−0.459, −0.232]	
	TS ⟶ PT	0.518	0.074	< 0.001	[0.367, 0.654]	70.96%

Indirect effects	TS ⟶ SE ⟶ PT	0.212	0.036	< 0.001	[0.149, 0.294]	29.04%

Total effects		0.730	0.052	< 0.001	[0.615, 0.823]	

*Note:* PT, presenteeism.

Abbreviations: SE, self‐efficacy; TS, transition shock.

## 4. Discussion

This study explored the situation of presenteeism among nursing students from China Medical University, Liaoning University of Traditional Chinese Medicine, and Shenyang Medical College in Shenyang, China. The survey results of this study showed that the presenteeism score of 421 Chinese nursing students was similar to the results of El‐Ashry AM et al.’s study on presenteeism among nurses [32]. This suggests that both employed nurses and nursing students have higher scores of presenteeism. The reasons for this may be that the interns during the transitional period are under pressure from multiple aspects [33, 34]. They have to cope with the high demands of theoretical learning, examinations, and clinical practice, leaving them physically and mentally exhausted. At the same time, they are subject to strict attendance rules, making it easy for them to engage in presenteeism; this may manifest as being physically present but cognitively absent, not actively thinking, not asking questions, and merely waiting for instructions [35]. Therefore, it is suggested that medical institutions and nursing managers should identify the current situation of nursing students as early as possible. They can train teaching instructors to recognize signs and observe the behavior of interns, such as avoiding eye contact, rarely speaking up voluntarily, and performing tasks mechanically and numbly [36]. Additionally, they should encourage interns to share experiences and implement multidimensional intervention measures to reduce the level of presenteeism among interns, thereby improving the quality of nursing talent cultivation.

In the multiple linear regression, we found that having a family member who is a medical worker, having served as a student cadre, and having an employment intention had a statistically significant positive impact on presenteeism. Gender had a statistically significant negative impact on presenteeism, that is, female students had lower presenteeism scores than male students. Notably, the attitude toward the nursing profession had a statistically significant negative impact on presenteeism. Compared with those with a good attitude toward the nursing profession, those with a general attitude had lower presenteeism scores. A reasonable explanation for this counterintuitive result might be that students with a good attitude toward the nursing profession have a higher sense of identity, mission, and responsibility toward the profession [37]. When they are physically unwell, they often choose to work while sick, that is, presenteeism, because they do not want to increase the burden on the team or adhere to the professional sense of responsibility to stay at their posts [38]. This finding does not deny their positive professional attitude but reveals a key mechanism: If individuals lack sufficient efficacy in coping with stress and self‐regulation, a good attitude toward the nursing profession may instead transform into a tendency toward presenteeism. Therefore, medical institutions and nursing managers need to pay attention to the psychological trait differences of nursing students and provide targeted support and intervention to more accurately prevent presenteeism and related negative consequences.

The foregoing analyses revealed the levels of three core variables and the predictors of presenteeism among nursing students, but these findings alone cannot explain the underlying pathways. To examine how transition shock influences presenteeism via self‐efficacy, we proposed four hypotheses (JD–R model) and tested them through correlation and mediation. The following sections discuss each in turn.

H1: Transition shock is positively correlated with presenteeism.

Our results confirmed that transition shock is significantly and positively correlated with presenteeism among nursing students, thus supporting Hypothesis 1. High‐intensity job demands deplete resources; when exhausted, nursing students resort to presenteeism due to insufficient energy to adjust or seek alternatives. Consistent with the JD–R model [39], stronger transition shock makes students more likely to ignore their health and continue working, thus worsening presenteeism.

H2: Transition shock is negatively correlated with self‐efficacy.

The study revealed a significant negative correlation between transition shock and self‐efficacy, supporting Hypothesis 2, which aligns with the findings of Sai Liang et al. [40]. Transition shock erodes nursing students’ clinical confidence and self‐efficacy through repeated failures, criticism, and workload. Given that self‐efficacy depends on mastery, vicarious experience, and emotions—all undermined by transition shock—mitigating this shock is key to preserving their self‐efficacy.

H3: Self‐efficacy is negatively correlated with presenteeism.

Our findings showed a significant negative relationship between self‐efficacy and presenteeism, confirming Hypothesis 3, which is consistent with the research of Qin Lin et al. [41]. Nursing students with higher self‐efficacy, more confident in coping with stress and illness, tend to adopt proactive health measures instead of working while ill. Conversely, those with lower self‐efficacy may overestimate the necessity of presenteeism, resulting in higher presenteeism. This highlights self‐efficacy as a protective personal resource.

H4: In nursing students, self‐efficacy plays a mediating role between transition shock and presenteeism.

The most critical finding is that self‐efficacy partially mediates the relationship between transition shock and presenteeism, thereby validating Hypothesis 4. This result is similar to those of existing studies [42, 43], indicating that transition shock not only directly and positively predicts presenteeism but also indirectly increases presenteeism by eroding self‐efficacy. Within the health impairment process of the JD–R model, this mediation result can be explained as follows. Transition shock—conceptualized as a high job demand—progressively consumes the limited cognitive and emotional resources of nursing students. In the face of role ambiguity and skill deficiencies during clinical internships, students are forced to expend substantial psychological resources simply to sustain routine performance, which inevitably erodes their self‐efficacy as a critical personal resource [44–46]. Once these personal resources are severely depleted and insufficiently replenished, individuals, in line with conservation of resources theory, incline toward maladaptive coping—reducing discretionary effort and avoiding challenging situations [47]. Presenteeism, defined as being physically present at work while psychologically disengaged, represents precisely such a defensive reaction to resource depletion [48]. Consequently, transition shock exerts its indirect effect on presenteeism through the depletion of self‐efficacy. This finding not only substantiates the applicability of the JD–R depletion pathway among nursing students but also identifies self‐efficacy as a pivotal psychological mechanism bridging environmental stress (transition shock) with a critical behavioral outcome (presenteeism).

Moreover, within the specific context of Chinese nursing education, nursing students simultaneously face high clinical workloads, academic pressures, and employment competition—triple stressors that amplify the erosive effect of transition shock on self‐efficacy and the occurrence of presenteeism [34]. Consequently, interventions should not be limited to behavioral control (e.g., mandatory rest) but should be advanced to the stage of psychological resource building. The core task for healthcare organizations and nursing managers is to buffer transition shock through structured support, such as positive feedback from clinical mentors and the establishment of a psychologically safe atmosphere, alongside regular self‐efficacy enhancement training. Effective strategies include designing stepwise clinical tasks, guiding interns to attribute successful experiences to their own efforts, and organizing peer experience‐sharing sessions [49, 50]. In sum, future educational practice should shift from “behavioral management” to “psychological empowerment.” Through systematic intervention, nursing students’ self‐efficacy can be strengthened, thereby enhancing their resilience to transition shock, reducing presenteeism, and facilitating their smooth development into proactive and competent professional roles.

### 4.1. Limitations

This study has several limitations. First, although the sample size met the requirements for mediation analysis, the generalizability of our findings is limited, as all participants were undergraduate nursing students from universities in Shenyang, China. Future research should employ a more diverse sample of nursing students (e.g., from other cities and institutions). Second, we used only the single‐item SPS‐6 scale without additional subjective or objective measures, which may not fully capture the multidimensional nature of presenteeism. Additionally, excluding participants with more than 7 days of sick leave resulted in a 100% prevalence rate under the dichotomous cutoff (> 6 scores); however, this finding reflects our sampling strategy rather than a clinical diagnosis, and we therefore used continuous scores in our analyses. Future studies should incorporate multiple assessment tools or objective indicators to further examine presenteeism. Third, although we found that self‐efficacy mediated the relationship between transition shock and presenteeism, other variables (e.g., social support) might also play a role in this pathway. Future research should explore other potential mediating variables to draw more robust conclusions. Finally, the cross‐sectional design precludes causal inferences. Future studies could adopt longitudinal designs or other methodologies to provide a stronger empirical basis for addressing presenteeism among nursing students.

## 5. Conclusion

The results of this study reveal that the impact of transition shock on the presenteeism of nursing students follows a dual path: a direct effect and an indirect effect mediated by self‐efficacy. This not only extends the application of the JD–R model to the internship context of nursing students but, more importantly, by identifying “self‐efficacy” as a key mediator, it points out a specific direction for formulating effective intervention measures to reduce presenteeism. We suggest that nursing managers not only focus on the identification and intervention of presenteeism but also consider the dynamic assessment of self‐efficacy. Timely implementation of intervention measures to enhance self‐efficacy is crucial for mitigating the negative impact of transition shock and reducing the level of presenteeism.

We recommend that medical institutions and nursing managers establish an assessment mechanism for the self‐efficacy of nursing students in practice, rather than merely focusing on the outcome indicator of presenteeism. Regular assessment should be conducted to promptly identify individuals with statistically significant fluctuations in self‐efficacy. This “monitoring—early warning—intervention” closed‐loop management can move the intervention point forward, stabilizing self‐efficacy before the negative impact of transition shock fully manifests as presenteeism, thereby fundamentally maintaining the effectiveness of clinical learning and improving the quality of talent cultivation in the nursing team.

## Funding

No fund or profit was received for this study.

## Ethics Statement

This study was approved by the Ethics Committee of Shengjing Hospital of China Medical University in Shenyang (2025PS1825K).

## Conflicts of Interest

The authors declare no conflicts of interest.

## Supporting Information

Additional supporting information can be found online in the Supporting Information section.

## Supporting information


**Supporting Information 1** This Supporting material contains one table (Table S1) presenting the fit indices of the structural equation model, including CMIN/DF, NFI, TLI, IFI, AGFI, CFI, GFI, RMSEA, and SRMR.

**Supporting Information 2** STROBE checklist_cross‐sectional.

## Data Availability

The data that support the findings of this study are available from the corresponding author upon reasonable request.
