# Supplementary material for: Mediating Role of Self‐Efficacy in Transition Shock and Presenteeism Among Nursing Students in China: A Cross‐Sectional Study
Source: J Nurs Manag. 2026 Sep 18;2026:5586956. doi: 10.1155/jonm/5586956 (PMC13589162; doi:10.1155/jonm/5586956)
Supplement: Supplementary file 1 [file JONM-2026-5586956-s001.docx]

**Table S1:The model fit of structural equation model.**

| **Fit Indices** | **Recommended Threshold** | | ****Model Fit**** | **Results** |
| --- | --- | --- | --- | --- |
|  | ****Excellent**** | **good** |  |  |
| CMIN/DF | ≤3 | ≤5 | 2.902 | **Excellent** |
| NFI | >0.9 | >0.8 | 0.965 | **Excellent** |
| TLI | >0.9 | >0.8 | 0.965 | **Excellent** |
| IFI | >0.9 | >0.8 | 0.977 | **Excellent** |
| AGFI | >0.9 | >0.8 | 0.933 | **Excellent** |
| CFI | >0.9 | >0.8 | 0.977 | **Excellent** |
| GFI | >0.9 | >0.8 | 0.964 | **Excellent** |
| RMSEA | ≤0.05 | ≤0.08 | 0.067 | Good |
| SRMR | ≤0.05 | ≤0.08 | 0.03 | **Excellent** |
